# The effect of cognitive-behavioral group therapy on reducing depression and anxiety in patients with mood disorders: experimental research

**DOI:** 10.1097/MS9.0000000000000971

**Published:** 2023-06-28

**Authors:** Pantea Nabian

**Affiliations:** Departmenta of Psychology, Tehran University, Tehran, Iran

**Keywords:** anxiety, cognitive-behavioral therapy group, depression, mood disorder

## Abstract

Mood disorders are one of the most common psychiatric disorders that manifest as a low mood in depressed people or a high mood in manic people. The cognitive-behavioral therapy group is one of the most effective forms of intervention available for patients with mood disorders. This research aimed to evaluate the effect of cognitive-behavioral group therapy in reducing depression and anxiety in patients with mood disorders.

**Case presentation:**

The study was a semi-experimental study with a pretest-post-test design with a control group. The research subjects were 60 patients hospitalized in the psychiatric department of Razi Hospital in Tehran, who were randomly divided into two experimental (*N*:30) and control (*N*:30) groups. Both groups took medicine as usual. Before the therapeutic intervention, both groups were evaluated with Beck’s depression questionnaire and the Zung anxiety scale. In addition to drug therapy, the experimental group participated in ten sessions of the cognitive-behavioral therapy group, and in the control group, no psychological intervention was performed except for drug therapy. At the end of the nonpharmacological treatment intervention, both groups were evaluated again with the aforementioned tests. The obtained data were analyzed using independent and dependent *t*-tests.

**Clinical discussion:**

The research findings showed that the cognitive-behavioral therapy group was significantly (*P*<0.05) effective in reducing the depression of hospitalized patients with mood disorders, but this method did not have much effect in reducing the anxiety of the patients.

**Conclusion:**

Cognitive-behavioral group therapy can be effective in reducing depression in hospitalized patients with mood disorders.

## Introduction

Highlights

- Mood disorders are one of the most common psychiatric disorders that manifest as low mood in depressed people or high mood in manic people.
- The cognitive-behavioral therapy group is one of the most effective forms of intervention available for patients with mood disorders.
- Cognitive-behavioral group therapy can be effective in reducing depression in hospitalized patients with mood disorders.

Mood disorders are among the most common psychiatric disorders that manifest as a low mood in depressed people or a high mood in manic people. In the etiology of this disorder, several factors have been mentioned, including biological, genetic, psychological, and social factors^1^. According to the etiology of this disorder, various treatment methods have been proposed. One of these approaches is cognitive-behavioral therapy, which has been proposed since the 1970s to treat all types of depressed patients, and its usefulness has been reported in several studies. In these studies, its therapeutic effect has been reported to be equal to or superior to antidepressant drugs^2–4^. Other studies have considered the effect of cognitive therapy to be equal to or superior to behavioral therapy^5,6^. Other studies have also shown that cognitive-behavioral therapy is more effective than supportive psychotherapy and analytical psychotherapy^7,8^.

In this method, the patient is encouraged to consider the relationship between negative thoughts and feelings of depression as hypotheses that should be tested and use the behaviors that lead to negative spontaneous thoughts as a benchmark to evaluate the validity or correctness of those thoughts. The cognitive-behavioral therapist tries to encourage the therapist to a kind of collaborative experience, and during it, he benefits from the patient’s own experiences in a series of behavioral experiments to evaluate the correctness or incorrectness of those beliefs^9,10^. In this approach, between 6 and 20 sessions of individual psychotherapy are needed for the treatment of depression until recovery occurs^11,12^.

Considering the prevalence of depressive disorder, which accounts for 75% of hospitalizations in mental hospitals, individual psychotherapy cannot be the answer to this problem. Therefore, the experts of this approach have tried to benefit from group therapy instead of individual psychotherapy, which is more economical in terms of cost and duration of treatment, with the title of cognitive-behavioral group therapy or logical-emotional group therapy^11,13^.

One of the important studies in the field of group therapy was conducted by Meichenbaum *et al.* ^14^. His emphasis was more on self-talk and he chose four treatment methods for groups^14^, which are as follows: insight into self-talk and its role in creating situational anxiety, mental desensitization, a combination of the two mentioned methods, and using a placebo.

After eight group therapy sessions in 8 weeks, he was evaluated at the end of the sessions and 3 months after that. Another study investigated the effect of the cognitive-behavioral therapy group in reducing depression and anxiety in patients with psychiatric disorders^15^. In evaluating the therapeutic achievements at the end and one year after the intervention, these researchers reported the usefulness of this method in the treatment of depression and anxiety.

In other research of the cognitive-behavioral therapy group for the treatment of women suffering from post-traumatic stress disorder, depressed patients used anger control caused by post-traumatic stress disorder, and reduced anxiety and depression in hospitalized patients with depression and reported it as useful^16^.

According to the mentioned materials, the study aimed to investigate the effect of cognitive-behavioral group therapy on reducing depression and anxiety in patients with mood disorders.

## Literature search

In a study titled the effect of the cognitive-behavioral therapy group of cognitive-behavioral skills on the symptoms of depression and anxiety of women with chronic schizophrenia, the results showed that there was a significant decrease between the two intervention and control groups in terms of changes in the average anxiety before and after the intervention. However, there was no significant difference in the average anxiety changes before and 6 months after the intervention and the average depression changes in the test stages^17^. In another study titled the effectiveness of cognitive-behavioral group therapy on death anxiety and general health of bereaved elderly women, the results showed that due to the high level of death anxiety and poor general health of bereaved elderly women, the findings of this research, the necessity of using educational therapy sessions Cognitive-behavioral to reduce death anxiety and improve the general health of the bereaved elderly^18^. In another study titled cognitive-behavioral therapy and its role in the outcome and recovery from schizophrenia, the results showed that although psychotherapy is the main line and the first line of treatment for schizophrenia, psychotherapy is an essential part of treatment for managing the remaining symptoms, inculcating social skills training, and disease management. Of the many nonpharmacological treatments, CBT is the most tested and relatively successful intervention in the management of schizophrenia^19^. In the study titled the effect of group cognitive-behavioral therapy to restore self-esteem on community members with mental illness, the results showed that from the intra-group trends and inter-group differences in self-esteem, we conclude that CBT may have a relatively long-term effect on restoring self-esteem .T2 is the turning point of emotions and cognition. Therefore, follow-up 3 months after the initial program is required^20^.

This study complements and strengthens the existing literature that CBT is effective in reducing the symptoms of patients with mood disorders and can be used to reduce the symptoms of these disorders.

## Method

The study was a semi-experimental study with a pretest-post-test design with a control group. Among the patients who were admitted to the men’s psychiatry department of Razi Hospital in Tehran, 60 met the conditions for entering the study and were randomly assigned to two experimental (*n*=30) and control (*n*=30) groups.

The first selected patients were evaluated with the help of the Beck depression test and the Zung anxiety scale. In addition to drug therapy, the patients of the experimental group participated in 10 group therapy sessions. But the patients of the control group were given only drug treatment and no type of psychotherapy was provided to them. The therapy group sessions were held two times a week for 5 weeks and the duration of each session was 1.5 h. The type of drug used in the experimental groups and evidence was mostly mood stabilizers (lithium carbonate, sodium valproate, and carbamazepine) and tricyclic antidepressants (mostly imipramine, nortriptyline, and trimipramine), and a percentage of patients from low and medium doses. They used antipsychotic drugs. After the end of the 10 sessions of the therapy group, both groups were evaluated with the help of the mentioned tests. The obtained data were analyzed by a *t*-test. Informed consent was obtained from all participants. The reporting guidelines were followed according to the study of Agha *et al.* ^21^. The protocol of each group psychotherapy session was developed with a cognitive-behavioral approach based on the available sources:

First session: In this session, the goals of group psychotherapy and the effect of group activity in solving problems were explained by the therapist. Then the patients introduced themselves and expressed their current discomfort, the reason for hospitalization, and the history of their illness.

The second meeting: At the beginning of the meeting, the opinions of the members regarding the previous meeting were examined. The symptoms of depression and its etiology were discussed in the group, and the therapist guided the group in such a way that they concluded that their way of thinking causes their emotions to be stimulated.

The third session: At the beginning of the session, their opinion about the previous session was asked. Then, there was a discussion about their unpleasant feelings, and an attempt was made to discover, with the help of the members, the self-negative thoughts along with the unpleasant feelings.

Fourth meeting: In this meeting, the members were introduced to the ABCD model, and its principles were taught to them.

A: Events that happened 1. B: Opinions about that event 2. C: Consequences of this way of thinking 3. D: Arguing with illogical thoughts 4 (negative spontaneous thoughts). At the end of each session, the members were asked to recognize the unpleasant feelings or negative spontaneous thoughts that come with them and try to engage with those thoughts and gradually replace them with positive or negative thoughts. In the fifth to tenth sessions, the main activity was focusing on the ABCD model and their behavioral activities and infrastructure assumptions.

## Tools

Zung anxiety status inventory: This scale was introduced by William Zung in 1974^22^ and is one of the most common clinical questionnaires about anxiety assessment. This scale has 20 questions that measure the symptoms and intensity of anxiety. Each question is graded in four levels. A grade of one is given if there is no symptom or its intensity is very low. A score of four is given in cases where there are always signs. As a result, the range of total scores of people fluctuates between 20 and 80. The alpha coefficient of this scale is reported to be 0.84, which indicates its internal stability. When the sample is limited only to patients who have been diagnosed with anxiety disorder, the amount of this correlation has also increased (r=0.74).

Beck Depression Questionnaire: This questionnaire was compiled by Beck in 1961 and revised in 1974. This questionnaire is one of the most widely used measurement tools for depression. Because many extensive studies have been done on the characteristics of psychometrics (reliability and validity) and its appropriate use^23^. Bumberry *et al.* ^24^ found that Beck scale scores in the student population were correlated with psychiatric interview ratings (r=0.77). In this study, the reliability of the test was 0.75 using the test-retest method, and its validity was 0.90 using the halving method. The average scores of depression and anxiety of the two experimental and control groups were analyzed before and after the therapeutic intervention with the help of an independent *t*-test.

## Results

The comparison of the mean depression scores in the two experimental and control groups in the stage before the implementation of the therapeutic intervention is presented in Table 1.

**Table 1:** Comparison of the average scores of depression and anxiety in two experimental and control groups before treatment.

Type of disease	Group	*N*	Mean	SD	df	*t*	.sig
Depression	Examination	15	35.08	12.91	12	0.87	0.43
	Control	15	30.06	9.63			
Anxiety	Examination	15	51.10	11.82	12	0.82	0.32
	Control	15	43.38	17.10			

As the Table 1 shows, there is no significant difference between the scores of the two groups. The comparison of the averages of the two groups in the Zung anxiety scale is not statistically significant. These results show that the two experimental and control groups did not differ significantly from the point of view of depression and anxiety before the intervention in the experimental group.

As the Table 2 shows, to investigate the effect of cognitive-behavioral group therapy in reducing patients’ depression, the mean difference between pretest and post-test depression scores in two groups was compared with the help of a *t*-test. As the table shows, the difference between the two groups is statistically significant (*P*<0.05). This finding confirms the first hypothesis of the research, which means that the subjects of the experimental group, who participated in the cognitive-behavioral therapy group in addition to drug therapy, showed more improvement in their depression level. To investigate the effect of the cognitive-behavioral therapy group in reducing anxiety, the mean scores of the Zung test of the two groups were compared with the help of the statistical *t*-test (Table 2). As Table 2 shows, the calculated *t*-score is not statistically significant. This finding shows that the two groups did not differ significantly from the point of view of the improvement of anxiety symptoms in the stage after the therapeutic intervention.

**Table 2:** Comparison of the mean difference of pretest and post-test depression and anxiety scores in the experimental and control groups.

Type of disease	Group	*N*	Mean	SD	df	*t*	.sig
Depression	Examination	15	17,68	5.12	12	4.12	0.06
	Control	15	8.52	8.19			
Anxiety	Examination	15	17.10	12.06	12	0.48	0.6
	Control	15	12.74	9.64			

## Discussion and conclusion

As stated, this research was conducted to investigate the effect of the cognitive-behavioral therapy group in reducing depression and anxiety in hospitalized patients with mood disorders. One of the hypotheses of this study was that the ‘cognitive-behavioral therapy group is effective in reducing depression in hospitalized male patients with mood disorders’. The comparison of the average difference between the scores of the Beck depression test in the pretest and post-test of the experimental group was statistically significant. Since the comparison of the average scores of the pretest of Beck depression in the two experimental and control groups did not show any difference in the pre-intervention phase, it can be concluded that there is a significant difference in the average difference between the pretest and post-test scores of the two groups only. In other words, the cognitive-behavioral group therapy method has been effective in reducing patients’ depression. This finding is consistent with the findings of other research conducted in the field of cognitive-behavioral therapy groups to reduce depression^25–29^.

In the mentioned studies, on average, the patients of each group participated in 12 sessions of cognitive-behavioral therapy group. In the present study, a 50% decrease in depression questionnaire scores was observed in the experimental group.

The second hypothesis was that ‘group therapy with a cognitive-behavioral approach is effective in reducing the anxiety of hospitalized male patients with mood disorders’. This hypothesis was not confirmed due to the mean difference of the Zung anxiety scale scores in the pretest and post-test in the experimental and control groups, which was not statistically significant, although the results show that the anxiety scores of both groups decreased in the post-test. One of the reasons that can be presented in this context is that the cut-off score on the Zung anxiety scale is 45 and 50 for the diagnosis of anxiety disorders. According to the average scores of this test (50.8 in the experimental group and 43.2 in the control group), maybe the anxiety level of the patients was not high enough to try to improve it.

The second reason may be that the patients were admitted to the ward with a diagnosis of depression, and in the process of group therapy, more emphasis was placed on the treatment of depression and less emphasis was placed on the treatment of anxiety. Of course, the effect of variables such as age, level of literacy, and duration of illness cannot be ignored.

## Conclusion

Group cognitive-behavioral therapy is due to the impact of depression during the follow-up to this point important that teach group cognitive-behavioral therapy techniques including relaxation, group cognitive-behavioral therapy of breathing techniques, and body-checking is equipped and teaching these techniques to depressed people. Cognitive-behavioral group therapy can be effective in reducing depression in hospitalized patients with mood disorders.

## Research limitations and suggestions

For more comprehensive studies on the effectiveness of cognitive-behavioral group therapy in the treatment of hospitalized depressed patients, it is suggested that future studies be conducted in centers where more depressed patients refer to them, so that the effect of education level, marital status, or it investigated the severity of depression in the effectiveness of group therapy, and in addition to the experimental and control groups, the placebo group should also be included in the study. In addition, in the present study, the presence of bipolar disorder in the patients was not evaluated, and this could have a major effect on the bias of the findings. Psychiatric diagnosis control is also suggested for more detailed research.

## Ethical approval

The authors assert that all procedures contributing to this work comply with the ethical standards of the relevant national and institutional committees on human experimentation and with the Helsinki Declaration of 1975, as revised in 2008. All procedures involving human subjects/patients were approved by [Research Ethics Committees of Imam Khomeini Hospital Complex- Tehran University of Medical Sciences.

## Consent

Written informed consent was obtained from the patient for publication of this case report and accompanying images. A copy of the written consent is available for review by the Editor-in-Chief of this journal on request.

## Sources of funding

None.

## Author contribution

P.N. designed the study, collected the data, analyzed the data, interpreted the results, and wrote and approved the final draft.

## Conflicts of interest disclosure

The authors declare no conflicts of interest.

## Research registration unique identifying number (UIN)

1. Name of the registry: NA.
2. Unique Identifying number or registration ID: NA.
3. Hyperlink to your specific registration (must be publicly accessible and will be checked): NA.

## Guarantor

Pantea Nabian.

## Data availability statement

Data are available from authors on request.

## Provenance and peer review

Not commissioned, externally peer reviewed.
